# Patterns of locoregional failure following post-operative intensity-modulated radiotherapy to oral cavity cancer: quantitative spatial and dosimetric analysis using a deformable image registration workflow

**DOI:** 10.1186/s13014-017-0868-y

**Published:** 2017-08-15

**Authors:** Abdallah S. R. Mohamed, Andrew J. Wong, Clifton D. Fuller, Mona Kamal, Gary B. Gunn, Jack Phan, William H. Morrison, Beth M. Beadle, Heath Skinner, Stephen Y. Lai, Sean R. Quinlan-Davidson, Abdelaziz M. Belal, Ahmed G. El-Gowily, Steven J. Frank, David I. Rosenthal, Adam S. Garden

**Affiliations:** 1 Departments of Radiation Oncology, The University of Texas MD Anderson Cancer Center, Unit 97, 1515 Holcombe Boulevard, Houston, TX 77030 USA; 2 Department of Head and Neck Surgery, The University of Texas MD Anderson Cancer Center, Unit 97, 1515 Holcombe Boulevard, Houston, TX 77030 USA; 3 Department of Clinical Oncology and Nuclear Medicine, Faculty of Medicine, University of Alexandria, Alexandria, Egypt; 4 Department of Clinical Oncology and Nuclear Medicine, Faculty of Medicine, University of Ain-Shams, Cairo, Egypt; 5 Department of Radiation Oncology, Allentown Radiation Oncology Associates, Allentown, PA USA

**Keywords:** Patterns of failure, Post-operative intensity modulated radiation therapy, Oral cavity cancer, Deformable image registration, Quantitative spatial and dosimetric analysis

## Abstract

**Background:**

We sought to identify spatial/dosimetric patterns of failure for oral cavity cancer patients receiving post-operative IMRT (PO-IMRT).

**Methods:**

Two hundred eighty-nine OCC patients receiving PO-IMRT were retrospectively reviewed from 2000 to 2012. Diagnostic CT documenting recurrence (rCT) was co-registered with planning CT (pCT) using a validated deformable image registration software. Manually segmented recurrent gross disease (rGTV) was deformed to co-registered pCTs. Mapped rGTVs were compared dosimetrically to planned dose and spatially to planning target volumes using centroid-based approaches. Failures types were classified using combined spatial/dosimetric criteria: A (central high-dose), B (peripheral high-dose), C (central intermediate/low-dose), D (peripheral intermediate/low-dose), and E (extraneous-dose).

**Results:**

Fifty-four patients with recurrence were analyzed; 26 local recurrence, 19 regional recurrence, and 9 both local and regional recurrence. Median time to recurrence was 4 months (range 0–71). Median rGTVs volume was 3.7 cm^3^ (IQR 1.4–10.6). For spatial and dosimetric analysis of the patterns of failure, 30 patients (55.5%) were classified as type A (central high-dose). Non-central high dose failures were distributed as follows: 2 (3.7%) type B, 10 (18.5%) type C, 1 (1.8%) type D, and 9 (16.7%) type E. Non-IMRT failure in the matching low-neck field was seen in two patients. No failures were noted at the IMRT-supraclavicular field match-line.

**Conclusions:**

Approximately half of patients with local/regional failure had non-central high dose recurrence. Peripheral high dose misses were uncommon reflecting adequate delineation and dose delivery. Future strategies are needed to reduce types C and E failures.

**Electronic supplementary material:**

The online version of this article (doi:10.1186/s13014-017-0868-y) contains supplementary material, which is available to authorized users.

## Introduction

Surgery is often the treatment of choice for oral cavity squamous cell carcinoma (OCSCC). Post-operative radiotherapy is indicated for OCSCC of advanced stages or with adverse prognostic factors [1–3]. Intensity-modulated radiotherapy (IMRT) enables conformal therapy and reduction of complications to surrounding normal tissue, and for many centers has become the standard radiation approach for head and neck cancer [4].

Generally, OCSCC patients demonstrate relatively worse loco-regional control compared to other head and neck subsites (e.g. oropharynx and larynx) [5–7]. Studies that have specifically examined cohorts of OCSCC patients receiving postoperative IMRT (PO-IMRT) have consistently reported only fair locoregional control rates, as low as 53% at 3 years in some series [8–13].

Moreover, most report failures as “infield”, “marginal”, or “outfield” based on percentage overlap between failure volume and respective target volumes. However, these studies applied non-uniform spatial methods for failure analysis, mainly utilizing non-validated rigid or manual image registration tools and without including the dosimetric component in the analysis [8–13]. We have recently shown the potential impact of patterns of failure analysis methodology using a validated image registration software paired with combined spatial and dosimetric analysis of failure, in improving the accuracy of reporting the patterns of failure in the era of IMRT [14–16]. As a continuation of these efforts we sought to apply this unique analytic methodology to our institutional large scale oral cavity cancer dataset of patients receiving PO-IMRT with documented treatment failure to achieve the following specific aims: 1) characterize distinct spatial and dosimetric patterns of failure after PO-IMRT, 2) identify clinical risk features associated with each failure type, 3) identify patterns of failure based target volume contouring recommendations, and 4) generate hypotheses for future clinical trials.

## Material and methods

### Patient selection

Two hundred eighty-nine patients with pathological diagnosis of OCC who received PO-IMRT at the University of Texas MD Anderson Cancer Center from 2000 to 2012 were retrospectively reviewed under an approved institutional review board protocol. Patients with distant metastases or concurrent malignancies at the time of diagnosis, or treatment with chemotherapy prior to staging at MDACC were excluded. Patients with prove of recurrence after PO-IMRT with available imaging documenting recurrence were included in the current analysis.

### Treatment planning and delivery

Treatment planning and delivery is described in details in previously published work, which examines the outcomes for this same patient cohort [17].

### Clinical data collection

Diagnostic contrast-enhanced CT and/or PET/CT documenting the initial evidence of local and/or regional recurrence (rCT) was identified. Recurrences were confirmed via radiologic imaging (i.e. progression in subsequent CT imaging or high SUV on PET imaging) or pathology specimens (i.e. from surgical biopsy). Radiologically evident recurrent gross disease (rGTV) was manually segmented and reviewed by two experienced radiation oncologists (ASRM, CDF). Corresponding original planning CTs (pCT) were also identified and original plans were restored. Patient, disease, and treatment characteristics were gathered during chart review.

### Image registration and dosimetric analysis

**Fig. 1:**
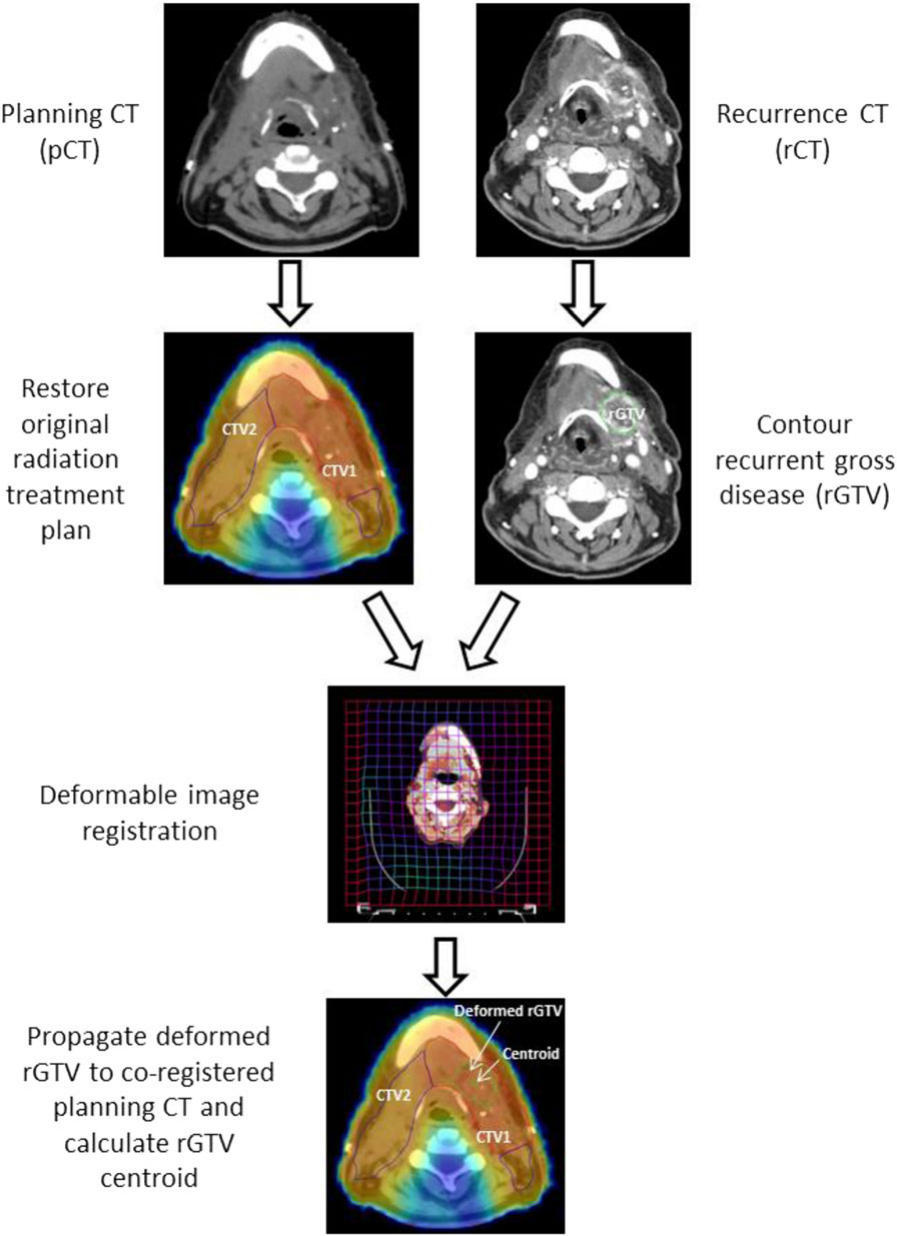
Workflow depicting how deformed rGTV is propagated to original planning CT

rCT was co-registered with pCT using a previously validated deformable image registration (DIR) methodology (VelocityAI 3.0.1, Velocity Medical Solutions, Atlanta, GA, 2004–2013) [14, 15]. rGTVs on the rCT were subsequently deformed to co-registered pCT (Fig. 1). The centroid, assumed as the origin of tumor recurrence, is represented as the calculated center of mass of the deformed rGTV. Dosimetric and volumetric parameters were obtained from the dose-volume histogram.

### Patterns of failure classification

**Fig. 2:**
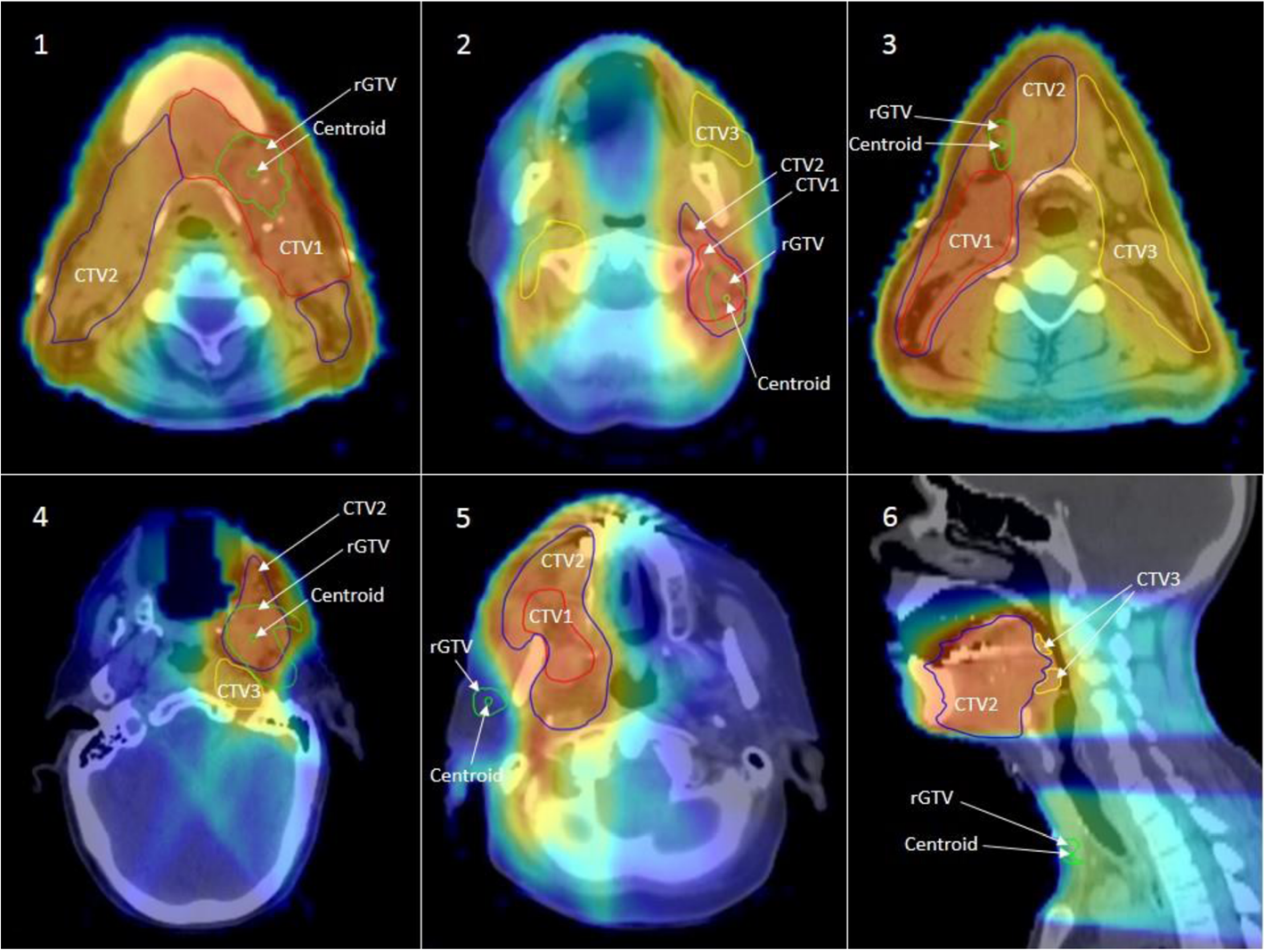
Examples of Failure Types. *1)* Type A (central high dose) failures. Centroid is mapped inside high dose TV and dose to 95% rGTV volume ≥ 95% dose prescribed to high dose TV. *2)* Type B (peripheral high dose) failure. Centroid is mapped inside high dose TV, but dose to 95% rGTV volume < 95% dose prescribed to high dose TV. *3)* Type C-int (central intermediate dose) failure. Centroid is mapped inside intermediate dose TV and dose to 95% rGTV volume ≥ 95% dose prescribed to intermediate dose TV. *4)* Type D-int (peripheral intermediate dose) failure. Centroid is mapped inside intermediate dose TV but dose to 95% rGTV volume < 95% dose prescribed to intermediate dose TV. *5*) Type E (Extraneous dose failure) where rGTV centroid originates outside all TVs. *6*) Type G (low neck failure) where rGTV centroid is located at the low-neck supraclavicular field

Failures are classified according to both spatial and dosimetric criteria as previously described. [16] Briefly, for spatial mapping of recurrence origin, the centroid of each rGTV was mapped to the corresponding TV in the planning CT. Subsequently, the dosimetric characteristics were assessed by calculating the dose to 95% of the failure volume (fD95%) then comparing it relative to the dose prescribed to the corresponding TV of origin as determined by the spatial mapping. Finally, failures were classified into five major types: Type A (central high dose where the mapped failure centroid originates in high dose TV and fD95% is ≥95% dose prescribed to corresponding high dose TV of origin), Type B (peripheral high dose where the failure centroid originates from high dose TV but its fD95% is <95% dose prescribed to corresponding high dose TV of origin), Type C (central elective dose where the failure centroid originates in lower dose TV and fD95% is ≥95% dose prescribed to corresponding lower dose TV of origin), Type D (peripheral elective dose where the failure centroid originates in lower dose TV but the fD95% is <95% dose prescribed to corresponding lower dose TV of origin), and Type E (extraneous dose where rGTV centroid originates outside all TVs). Type F describes junctional failures at the IMRT/supraclavicular match line, and Type G describes low neck failures at the low-neck supraclavicular field. Type G is analogous to type C if the fD95% is ≥95% dose prescribed to the low-neck and analogous to type D if the fD95% is <95%. Examples demonstrating failure type definitions are illustrated in Fig. 2.

Patients were then classified according to the predominant mode of failure. Patients with more than single recurrence lesion were classified as the following: 1) for patients with type A recurrence and concurrent non-type A lesions, the overall pattern of failure was defined as type A because we believe type A for such patients is the true recurrence rather than reseeding from the non-type A recurrence, 2) for patients whom exhibited more than one failure type of non-type A simultaneously, pattern of failure of each patient was classified according to the predominant type as determined by the most commonly encountered failure type (i.e. higher number or higher volume).

## Results

### Patient and treatment characteristics

Sixty-three patients (22%) developed locoregional recurrences. Median time to recurrence was 4 months (range 0–71). For spatial and dosimetric analysis of the patterns of failure, 9 patients were excluded: 4 with post-surgical recurrence prior to initiation of IMRT, 3 with no retrievable IMRT plan, and 2 with no available imaging documenting recurrence. This left 54 patients for the current analysis.

Patient, disease, and treatment characteristics for the analyzed 54 patients are summarized in Table 1. The most common primary site was the oral tongue (39%). The most common pathologic T and N staging were T2 (37%) and N2 (36%). Forty-seven (87%) patients had Stage III-IV disease.

**Table 1:** Patient, disease, and treatment characteristics

Characteristic	N (%)
Age	
Median (range)	59.5 years (22–87)
Gender	
Female	20 (37)
Male	34 (63)
Tumor Site	
Oral Tongue	21 (39)
Buccal Mucosa	10 (18.5)
Floor of Mouth	2 (4)
Hard Palate	3 (5)
Gingiva	10 (18.5)
Retromolar Trigone	8 (15)
Histologic Differentiation	
Poor	13 (24)
Moderate	36 (67)
Well	5 (9)
Clinical T stage	
T1	8 (15)
T2	19 (35)
T3	9 (17)
T4	18 (33)
Clinical N stage	
Nx	2 (4)
N0	23 (43)
N1	11 (20)
N2a	0 (0)
N2b	14 (26)
N2c	4 (7)
Pathological T stage	
ypT0	2 (4)
T1	8 (15)
T2	20 (37)
T3	7 (13)
T4	17 (31)
Pathological N stage	
No dissection	7 (13)
N0	12 (22)
N1	12 (22)
N2a	0 (0)
N2b	19 (36)
N2c	4 (7)
Overall stage	
Stage I	1 (2)
Stage II	6 (11)
Stage III	7 (13)
Stage IV	40 (74)
Primary Surgery Margin Status	
Negative (>5 mm)	41 (76)
Close (≤ 5 mm)	9 (17)
Positive	4 (7)
Depth of invasion	
≤ 1.5 cm	33 (61)
> 1.5 cm	18 (33)
Unspecified	3 (6)
Perineural invasion	
Yes	22 (41)
No	32 (59)
Lymphovascular invasion	
Yes	14 (26)
No	30 (56)
Unspecified	10 (18)
Extracapsular extension	
Yes	17 (31)
No	37 (69)
IMRT dose and fractionation	
Median Dose (Range), in Gy	60 (56–70)
Median Fractionation (Range)	30 (28–33)
Laterality of Neck radiation	
Unilateral	13 (24)
Bilateral	41 (76)
Chemotherapy	
Induction	5 (9)
Concurrent	13 (24)
Induction and concurrent	2 (4)
No chemotherapy	32 (59)

Surgical margins were positive and close (defined as ≤5 mm) in 4 (7%) and 9 (17%) patients, respectively. Perineural invasion or lymphovascular invasion was present in 22 (41%) and 14 (26%) patients, respectively. Depth of invasion was ≥1.5 cm in 18 patients (33%). Forty-seven patients (87%) had neck dissections, and of those 17 patients had nodal extracapsular extension.

Mean RT dose was 60 ± 7 Gy and mean number of fractions was 30 ± 3. One patient did not complete the full course of IMRT, discontinuing therapy after 6 fractions. Thirteen (24%) and 41 (76%) patients received unilateral and bilateral neck irradiation, respectively. Mean overall treatment package time, defined as time interval from date of surgery to last day of irradiation, was 12.3 ± 1.7 weeks.

### Recurrence characteristics

**Fig. 3:**
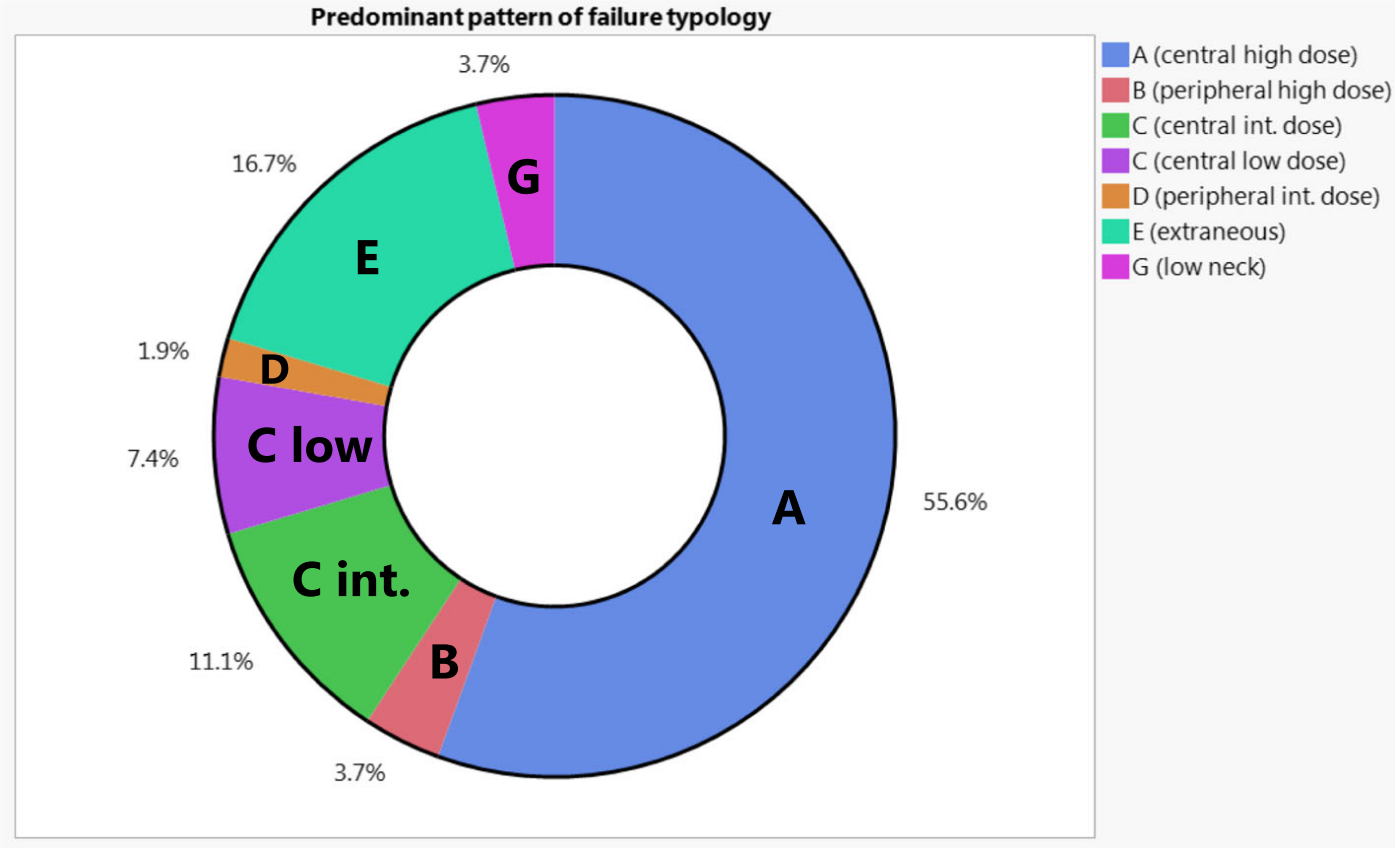
Ring chart that depicts the distribution of the predominant typology of failure for the entire dataset (*n* = 54)

For patients included in the current analysis; 26 (48%) had local recurrence, 19 (35%) had regional recurrence, and 9 (17%) had both local and regional recurrence. A total of 82 rGTVs were delineated. Median rGTVs volume was 3.7 cm^3^ (IQR 1.4–10.6). Figure 3 shows the distribution of the predominant type of failure for the entire dataset using the proposed classification schema. Thirty patients (55.5%) were classified as type A. Non-type A failures were distributed as follows: 2 (3.7%) type B, 10 (18.5%) type C, 1 (1.8%) type D, 9 (16.7%) type E, and 2 (3.7%) type G. Because type A “central high dose” failures, are considered to resistance to maximal therapy, and thus could not conceivably be prevented by technical/operator dependent processes, however, non-type A failures represent a major goal for IMRT quality assurance and improvement. Table 2 illustrates the details of the characteristics for all non-type A failures.

**Table 2:** Failure Sites for non-type A Failures

Patient	Local vs Regional	Predominant Failure Type	Primary Tumor Site	Pathologic TN Stage	Surgical margins	Neck dissection status and laterality	ECE	Neck Irradiation Laterality	DOI	PNI	LVI	Chemotherapy	CTV1 Dose (Gy/n. of Fracions)	Failure Site
1	L	B (peripheral high dose)	gingiva	T2 N0	-ve	-ve Ipsilat	-ve	Ipsilat	≤1.5 cm	+ve	-ve	No	60/30	Ipsilat RMT&Maxilla
2	L	B (peripheral high dose)	FOM	T1N2c	-ve	+ve Bilat	+ve	Bilat	>1.5 cm	-ve	-ve	No	13/6^b^	FOM
3	L	C (central int. dose)	RMT	yT4ayN0	-ve	-ve Ipsilat	-ve	Bilat	≤1.5 cm	-ve	U/S	I + C	60/30	Ipsilat Masticator Space
4	L	C (central int. dose)	tongue	T4aN2b	-ve	+ve Bilat	-ve	Bilat	>1.5 cm	-ve	-ve	No	60/30	FOM
5	L	C (central int. dose)	FOM	T4aN1	-ve	+ve Bilat	-ve	Bilat	>1.5 cm	-ve	-ve	No	60/30	BOT
6	L	A^a^	RMT	T4aN1	+ve	+ve Ipsilat	-ve	Ipsilat	>1.5 cm	+ve	-ve	No	70/33	Flap recurrence + Ipsilat Parotid & Masticator Space
7	L	D (peripheral int. dose)	gingiva	T4aN1	+ve	+ve Ipsilat	-ve	Bilat	≤1.5 cm	+ve	+ve	No	63/30	Ipsilat Masticator Space
8	L	E (extraneous)	FOM	T1 N1	-ve	+ve Bilat	-ve	Bilat	≤1.5 cm	+ve	+ve	No	60/30	Mandibular gingiva
9	L	E (extraneous)	gingiva	T4aN1	-ve	+ve Ipsilat	-ve	Ipsilat	≤1.5 cm	-ve	-ve	No	60/30	Contralat mandibular gingiva
10	L	E (extraneous)	gingiva	T4aN2b	close	+ve Ipsilat	-ve	Ipsilat	≤1.5 cm	+ve	-ve	C	60/30	Ipsilat masticator space
11	L	E (extraneous)	Buccal	yT2yN0	-ve	-ve Ipsilat	-ve	Ipsilat	>1.5 cm	-ve	-ve	I + C	60/30	Ipsilat perineural spread along V2&V3
12	LR	C (central int. dose)	tongue	T1 N1	-ve	+ve Ipsilat	+ve	Bilat	≤1.5 cm	-ve	-ve	C	60/30	FOM&Contralat level IIa
13	LR	C (central low dose)	tongue	T2 N0	-ve	-ve Ipsilat	-ve	Bilat	≤1.5 cm	-ve	+ve	C	64/30	Contralat tongue&level II
14	R	C (central low dose)	Hard palate	T3 N0	-ve	-ve Ipsilat	-ve	Bilat	≤1.5 cm	-ve	-ve	No	60/30	Contralat level IIa
15	R	C (central low dose)	gingiva	T4aNx	-ve	No dissection	N/A	Ipsilat	≤1.5 cm	-ve	-ve	No	64/32	Ipsilat level Ib
16	R	C (central int. dose)	RMT	T2 N1	-ve	+ve Ipsilat	-ve	Ipsilat	≤1.5 cm	-ve	-ve	No	60/30	Ipsilat level Ib
17	R	C (central low dose)	tongue	T2 N1	-ve	+ve Ipsilat	-ve	Bilat	≤1.5 cm	-ve	-ve	No	60/30	Contralat level Ib
18	R	C (central int. dose)	tongue	T1 N1	-ve	+ve Ipsilat	-ve	Bilat	≤1.5 cm	-ve	-ve	No	56/28	Level Ia
19	R	A^a^	tongue	T3N2b	-ve	+ve Ipsilat	+ve	Bilat	>1.5 cm	+ve	+ve	C	60/30	Ipsilat level III (A) Contralat level IIa (C)
20	R	A^a^	RMT	T4aN2b	close	+ve Ipsilat	-ve	Bilat	≤1.5 cm	-ve	U/S	C	60/30	Ipsilat level IIb (A) Ipsilat retropharyngeal node (D)
21	R	E (extraneous)	Buccal	yT3yN0	close	-ve Ipsilat	-ve	Ipsilat	≤1.5 cm	+ve	-ve	I	60/30	Contralat Pterygoid plates& maxilla
22	R	E (extraneous)	Buccal	T2N2b	close	+ve Ipsilat	-ve	Ipsilat	≤1.5 cm	-ve	+ve	No	60/30	Ipsilat parotid node
23	R	E (extraneous)	Buccal	T2N2b	-ve	+ve Ipsilat	+ve	Ipsilat	≤1.5 cm	-ve	-ve	C	60/30	Ipsilat parotid node
24	R	E (extraneous)	Buccal	T1N2b	close	+ve Ipsilat	+ve	Ipsilat	U/S	-ve	U/S	C	63/30	Contralat levels II, III, IV
25	R	E (extraneous)	gingiva	T2Nx	+ve	No dissection	N/A	Ipsilat	≤1.5 cm	-ve	U/S	No	65/30	Contralat level II
26	R	G (low neck)	tongue	T1N2b	-ve	+ve Ipsilat	-ve	Bilat	≤1.5 cm	+ve	+ve	No	60/30	contralat levels III, IV
27	R	G (low neck)	gingiva	yT4ayN0	close	-ve Ipsilat	-ve	Ipsilat	>1.5 cm	+ve	U/S	I	60/30	Ipsilat level VIb

^a^Indicates type A failure with multifocal recurrence that includes non-type A lesions as well. ^b^This patient had received only 6 fractions and failed to appear for the remainder of her treatments. *Abbreviations*. *ECE *extracapsular extension, *DOI* depth of invasion, *PNI *perineural invasion, *LVI *lymphovascular invasion, *L* local, *R* regional, *LR* locoregional, *FOM* floor of mouth, *RMT* retromolar trigone, *−ve* negative, *+ve* positive, *Ipsilat* ipsilateral, *Contralat* contralateral, *Bilat* bilateral, *U/S* unspecified, *I* induction chemotherapy, *C* concurrent chemotherapy, *I + C* induction followed by concurrent chemotherapy, *No* no chemotherapy

### Local failure typology

Of the 26 patients with local disease failure without synchronous regional recurrence, 16 (62%) were type A central high dose failures. Ten patients (38%) had non-type A local failure; two (8%) were type B, three (11%) were type C (intermediate dose), one (4%) was type D (intermediate dose), and four (15%) were extraneous type E failure. Three of patients with type A failure had multifocal recurrence; two patients with two foci of recurrences (both within the central high dose region), and one had four foci of recurrences (three in the intermediate dose and one in the high dose).

For the two patients with type B failure, one had a primary tumor in the left mandibular gingiva and developed recurrence involving the left maxillary sinus, with erosion of its lateral wall and extension along the buccal mucosa to the retromolar trigone. The second patient had a primary tumor in the floor of mouth with rapid disease progression subsequent to discontinuing radiation treatment after six fractions.

Regarding patients with type C failure, one patient had his primary tumor in the retromolar area and recurrence in the ipsilateral masticator space. The second patient had the primary tumor in the floor of mouth with recurrence involving the base of tongue, while the third patient had a primary tumor in the oral tongue with recurrence involving the floor of mouth.

The single type D failure had the primary tumor in the left mandibular gingiva and recurred along the left masticator space allowing the rGTV to partially grow outside the CTV2 boundaries, however the centroid was still located inside CTV2.

**Fig. 4:**
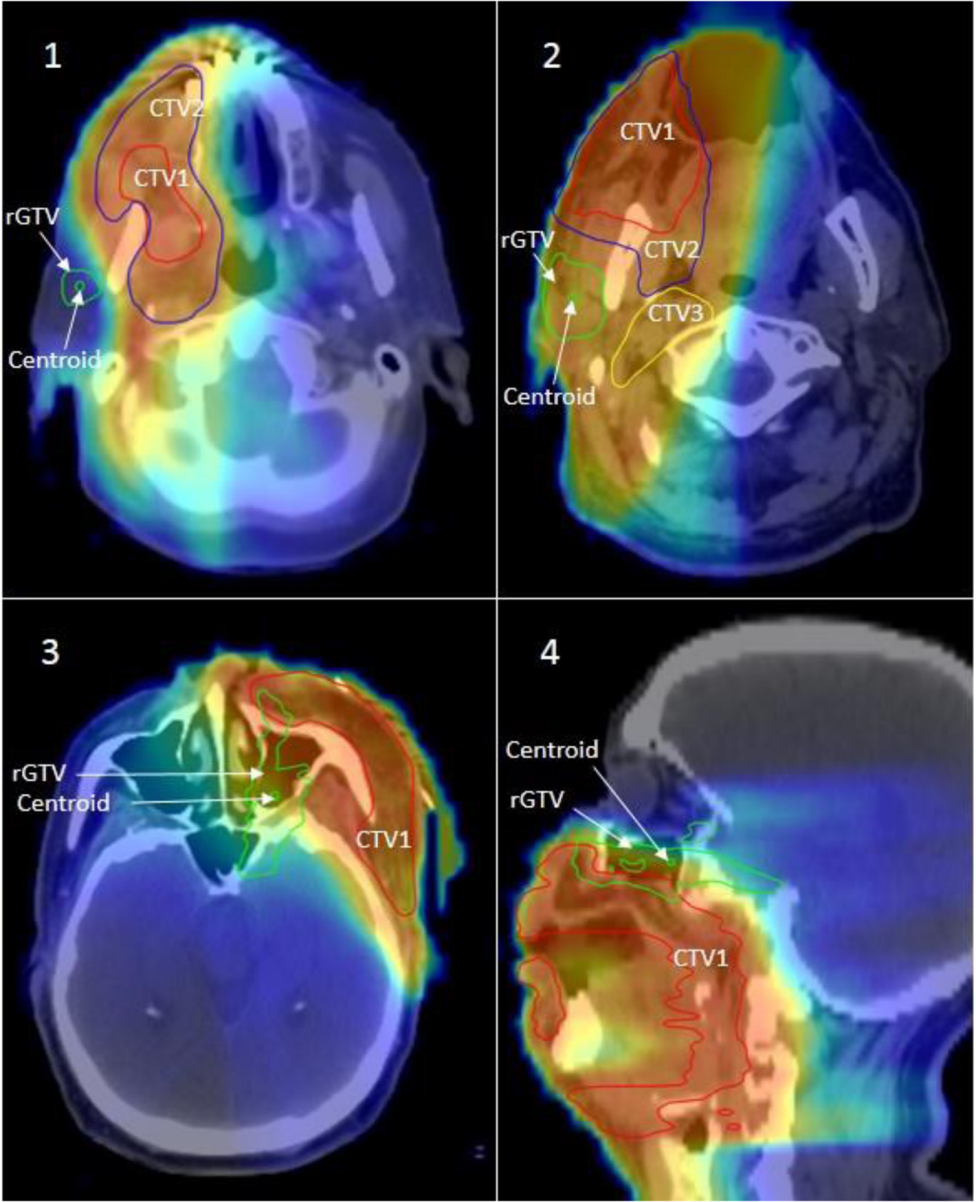
The top panel depicts two patients with type E recurrence in the ipsilateral parotid nodal area following parotid sparing IMRT. Both patient*s* were diagnosed with T2N2b right sided buccal mucosa primaries and subsequently failed at the ipsilateral parotid area outside all target volumes. The lower panel depicts another example of type E failure in a patient diagnosed with T3N2b at the buccal mucosa with post-IMRT ipsilateral perineural spread along the maxillary and mandibular nerves (*bottom left, bottom right)*

For patients with type E failure, one had the primary tumor in the right mandibular gingiva and the recurrence in the contralateral mandibular gingiva approximately 2 years following treatment. The second patient had the primary tumor in the left buccal mucosa; the recurrence manifested as retrograde perineural spread that extended to the left pterygopalatine fossa, foramen rotundum, foramen ovale, cavernous sinus, and through the superior orbital fissure into the left orbit (Fig. 4). The third patient had T1 primary tumor in the central floor of mouth and the recurrence in the left alveolar mandibular ridge approximately 3 years following treatment. Lastly, the fourth patient had the primary tumor in the left mandibular gingiva and the recurrence in the ipsilateral masticator space at the first follow up following treatment.

### Regional failure typology

Of the 19 patients with regional disease failure without synchronous local recurrence, only 7 patients (37%) were type A central high dose failures. Twelve patients (63%) had non-type A local failure; 5 (26%) were type C (intermediate or low dose), five (26%) were extraneous type E, and 2 (11%) were type G low neck failure. Two of patients with type A failure had multifocal recurrence. One patient with a left oral tongue primary developed synchronous ipsilateral type A recurrence at level III and contralateral type C (low dose) recurrence at level IIa. The second patient had the primary disease in the right retromolar trigone with multi-nodal recurrence at ipsilateral neck level IIb and an ipsilateral retropharyngeal lymph node (type D).

For the five patients with type C failure, one patient had a right hard palate primary with multifocal type C (low dose) failure with two foci of recurrence, both at contralateral level IIa. The second patient had the primary tumor in the left maxillary ridge and recurred in the low dose region at the ipsilateral level Ib. The third and fourth patients had primary tumors of the oral tongue with recurrences in levels Ia (intermediate dose) and contralateral Ib (low dose), respectively. The fifth patient had a primary tumor in the right retromolar trigone and recurrence in the ipsilateral level Ib (intermediate dose).

Four of the five patients with type E extraneous failure had their primary tumor in the buccal mucosa. Two patients had ipsilateral parotid nodal recurrence in the area of spared parotid gland (Fig. 4) while the other two patients had recurrences in the un-irradiated contralateral side. The fifth patient had the primary tumor in the left maxillary alveolar ridge with recurrence in the un-irradiated contralateral level II.

Regarding the two patients with type G low neck failure, one had the primary tumor in the left mandibular with negative dissection of ipsilateral neck levels II, III, and IV, however, the patient then recurred in the ipsilateral level VIb (i.e. pre-tracheal recurrence). This failure is analogous to type D as fD95% for this patient was 10 Gy (i.e. fD95% had less than 95% of the dose prescribed to left supraclavicular region which was 58 Gy). The second patient had the primary tumor in the left oral tongue with positive neck dissection of ipsilateral levels I, II, and V then recurred in the contralateral levels III and IV. This failure is analogous to type C as fD95% for this patient was 48.75 Gy (i.e. fD95% had higher than 95% of the dose prescribed to contralateral supraclavicular field that encompassed both contralateral levels III and IV with a prescription dose of 50 Gy).

### Locoregional failure typology

Nine patients had synchronous local and regional failure. The predominant typology for each patient was determined according to the local failure component. Seven (78%) had type A failure and two (22%) had type C. For patients with type A failure, 3 had synchronous non-type-A failure at the neck (2 had synchronous type C and one had synchronous type G). Both patients with type C failure had their primary tumor in the oral tongue. The first had recurrence in the contralateral side of the tongue and neck level II (both at low dose CTV) while the second had recurrence in the floor of mouth (intermediate dose CTV) and contralateral neck level IIa (low dose CTV).

## Discussion

Our results demonstrate that nearly half of the patients with local and/or regional failure included in the current study had non-central high dose recurrence. We applied our novel patterns of failure analysis and classification methods to further analyze those recurrences of non-central high dose nature. Recurrences in operated tissues are less prone to present concentrically as do recurrences that occur following definitive radiation. The likelihood of a recurrence manifesting in the epicenter of origin disease that is removed surgically is unlikely, particularly when large volumes of tissue are removed and replaced by free flaps, creating significant variations in the irradiated anatomy compared to the presurgical anatomy. Furthermore, the new tissue planes can create differing paths for tumor to spread through. Thus in the postoperative setting we essentially create a crude probabilistic model of where microscopic disease may be hiding.

While concentric central recurrences occurred less commonly than we noted in the definitive setting, over 75% of recurrences did occur within targeted tissues, the majority of which were in the high dose tumor bed target (Type A), while the remainder were in subclinical (Type C and G) targets. No technical or operator-dependent processes could conceivably prevent such failures. Whether dose intensification to Type A targets would minimize these recurrences is unclear. To date, the benefits of treatment intensification seem small. There is a paucity of data demonstrating that increasing radiation dose is beneficial, and even concurrent chemoradiation only seems to benefit those at highest risk [5, 18]. Type C failures may be prevented by prescribing higher doses (i.e. shifting to higher TV levels), but it is unclear if these relatively small dose increments (as the differences between dose to each CTV was <10%) would be beneficial, and also increasing dose to larger volumes can potentially increase the risk: benefit ratio. Non-IMRT failure in the matching low-neck supraclavicular field was very uncommon and only seen in two patients. Also, no failures were noted at the IMRT-supraclavicular field match-line confirming the safety of this technique.

Types B (peripheral high dose), D (peripheral elective dose) and E (extraneous) failure are potentially dependent on technical or radiotherapy processes. Type B and D recurrences are analogous to what has been described as “marginal miss”. Peripheral misses (type B) were seen only in 4 patients (of whom one didn’t complete the prescribed radiation dose). Three of these were in primary tumor sites, and more likely reflect the tumors finding pathways more amenable for spread, and growing out of the dose region rather than originating at the periphery. Similarly, the one peripheral nodal failure was in a retropharyngeal node that is typically not targeted, but fell into a D type failure rather than E due to the proximity to the primary tumor bed. The paucity of these peripheral type recurrences reflects an adequate CTV delineation strategy, appropriate PTV margins, and precise dose delivery.

Type E extraneous recurrences were seen in approximately 17% of failures. Type E failures are analogous to traditionally defined “out of field” recurrences. This pattern of failure was mainly in patients who had primary tumors of the buccal mucosa or gingiva. Four patients had primary type E recurrence, and 5 had nodal type E recurrences. Two patients with type E failure at the primary site had recurrences in sites relatively separate from the primary disease, and so these “recurrences” may represent second primary tumors.

Retrograde ipsilateral perineural spread was the cause of Type E recurrence in two patients (Fig. 4), and was also observed in two other patients (1 Type C and 1 Type D) as seen in Table 2. Daly et al. had reported on a patient who had developed failure at the ipsilateral masseter due to presence of perineural invasion and retrograde tracking along the mandibular nerve [11]. Yao et al. had previously reported on two patients with extensive perineural invasion and retrograde tracking who had developed failure within the infratemporal fossa [8]. We would also recommend that “nerves at risk in the tumor bed should undergo biopsy and be covered in a retrograde fashion within the RT field” [11].

Three patients had recurrence in the contralateral undissected/unirradiated upper neck. Prior studies have reported that positive ipsilateral lymph nodes are a predictive factor for contralateral recurrence; conversely, contralateral lymph node metastases never occurred in patients without ipsilateral lymph node involvement [19, 20]. While these studies demonstrated the association of ipsilateral lymph node involvement with contralateral recurrence, the majority of patients in these studies were predominantly patients with oral tongue cancer, and few patients had buccal cancer. We continue to favor comprehensive bilateral radiation for patients with tumors in central oral cavity sites, such as the oral tongue and floor of mouth. Yao et al. recommended that patients with ipsilateral lymph node involvement in OCC should receive bilateral neck irradiation [8]. Moreover, Chan et al. suggested that bilateral neck irradiation should be administered to patients with N2b disease [13]. However, again these studies were heavily weighted with patients with oral tongue, and not buccal cancers. Thus our approach to buccal and retromolar trigone tumors is individualized. Those patients with low nodal burden are still treated to the ipsilateral neck, but those with bulky nodes, multiple levels of nodes, or who have an epicentered lateral primary site but the primary disease extends centrally are treated to both sides of the neck.

Two patients had almost identical pattern of recurrence in the ipsilateral parotid nodes as shown in Fig. 4. Strict dose constraints to the parotid have been recommended to avoid long-term risks of xerostomia [10]. In our cohort, two patients with T2N2b buccal mucosa primaries had recurrences in the ipsilateral parotid gland which was spared during PO-IMRT. This phenomena has been also reported in previous studies [13]. The proximity of buccal and retromolar trigone tumors to the parotid bed makes ipsilateral parotid avoidance challenging. We therefore recommend limiting the extent of radiation-induced xerostomia by focusing on sparing the contralateral glands.

To date, a limited number of studies have exclusively investigated failure following PO-IMRT in OCC patients. Additional file 1: Tables S1 and S2 tabulate our study’s patient/treatment and failure characteristics compared to extant literature. Loco-regional control of our study are consistent with that of previous studies [8–13, 21]. Although Sher et al. reported low loco-regional failure rates (7%), they acknowledged that it may have reflected the greater proportion of early T and N staging in their cohort [12]. Other disease characteristics are noted but are not directly comparable as the subset of reported patients varied from study to study.

To classify failures using a spatial component, several prior studies [8, 9, 11–13] used varying volume overlap approaches [22–24]. Here we highlight two limitations in these prior studies: 1) volume overlap methods for spatial characterization and 2) the lack of a dosimetric component in failure analysis. Given enough time, recurrence volumes can outgrow TV margins. Thus, the spatial characterization of “infield” vs. “outfield” is volume dependent and biased by elapsed time. As the spatial component of our failure classification, we apply a centroid-based approach. This approach has demonstrated to be more superior and accurate than volumetric overlap approaches, as the latter tends to assign failures more peripherally [25, 26]. Moreover, the spatial component alone is insufficient for accurate and specific reporting of failures. Without a dosimetric component, failures that are “infield” but in fact did not receive the prescribed dose (i.e. Types B and D in our classification) could be erroneously assumed to be biological failures. Subsequently, such “infield” failures are not investigated further despite a potentially rectifiable technical or radiotherapy process.

As a retrospective series, the standard caveats apply. However, although patients were not selected nor treated prospectively, all patients were reviewed by a multidisciplinary team. This data was collated as a secondary analysis of part of a larger programmatic evaluation of PO-IMRT outcomes for OCC; the reader is encouraged to peruse the clinical/oncologic report previously published [17]. Likewise, as a single institute series from a high-volume tertiary center, the generalizability/scalability of our findings to facilities which do not utilize our systematic quality assurance methods (e.g. multi-physician direct physical examination and consensus review of target delineation) would be suspect [27, 28].

Nevertheless, despite these limitations, our study is the largest, to our knowledge, systematic assessment of patterns of failure to OCC following PO-IMRT using a quality-controlled image-registration pipeline for methodologically rigorous pattern of failure investigation [16]. Similarly, our study is the first to incorporate a dosimetric component in failure classification for OCC following PO-IMRT, in addition to utilizing a centroid-based spatial component and a validated DIR method which is critical for accurate failure analysis [14–16]. We hope that by utilizing a standardized typology for reporting patterns of failure in OCC following PO-IMRT, which can be adopted by multiple institutions, we can encourage other comparable reporting practices for PO-IMRT, in a manner that allows improved detection of possible modes of preventable error. This could allow for pooling of data to infer differences in treatment approaches and subsequent outcomes amongst different institutions.

## Conclusions

Prior studies have assessed loco-regional control following PO-IMRT to OCC in manner which elides the reality of dosimetric gradients inherent in IMRT, and precludes identification of systematic sources of modifiable error which might impact these recurrences. A standardized typology with both spatial and dosimetric components allows for more accurate and specific reporting of the patterns of failure over traditional “infield” vs. “marginal” vs. “outfield” failure classification schemes. Our study incorporates a dosimetric component in addition to utilizing a centroid-based spatial component and a quantitatively validated DIR method. Approximately half of the patients with local and/or regional failure included in the current study had non-central high dose recurrence. Thus, contrary to non-OCC sites, a substantial proportion of failures in our series, despite rigorous multiphysician quality assurance, are not definitive biological failures and, as potentially modifiable risk-events, necessitate further investigation and potential practice modification. Other groups are encouraged to undertake similar efforts as single-site or pooled analyses for OCC following PO-IMRT.

## Additional file

Additional file 1:Tables S1 and S2. Showing our study’s patient/treatment and failure characteristics compared to extant literature. (PDF 99 kb)

## Abbreviations

DIR: Deformable image registration
fD95%: dose to 95% of the failure volume
IRB: Institutional review board
pCT: planning CT
PO-IMRT: Postoperative intensity modulated radiotherapy
rCT: recurrence CT
rGTV: recurrence gross tumor volume
ROIs: Regions of interest
TVs: Target volumes
