# Supplementary material for: Patterns of locoregional failure following post-operative intensity-modulated radiotherapy to oral cavity cancer: quantitative spatial and dosimetric analysis using a deformable image registration workflow
Source: Radiat Oncol. 2017 Aug 15;12:129. doi: 10.1186/s13014-017-0868-y (PMC5557312; doi:10.1186/s13014-017-0868-y)
Supplement: Additional file 1:Tables S1 and S2. — Showing our study’s patient/treatment and failure characteristics compared to extant literature. (PDF 99 kb) [file 13014_2017_868_MOESM1_ESM.pdf]

Supplementary tables

Supplementary table 1: Published Reports Describing Patterns of Failure Following Post-Operative IMRT for Oral Cavity Cancer, Patient Characteristics

Author	Time period	Patients with PO-IMRT for OCC (n)	LRC (%,at x years)	Patients with LRF following PO-IMRT for OCC (n,%)	Median RFS in months (range)	T3-T4 (%)	pN+ (%)	Positive margin (%)	ECE (%)	PNI (%)	LVI (%)	CRT (%)
Yao et al. (9)	2001-2005	49	82 (2y)	8 (16%)	4.1 (3.0-12.1)	56[a]	69[a]	NR	NR	NR	NR	4[b]
Studer et al. (10)	2002-2007	28	92 (2y)	NR	NR	32[b]	75[b]	NR	NR	NR	NR	85[b]
Gomez et al. (11)	2000-2006	35	84 (2y)	6 (17%)	6.1 (4.1-26.0)	40[b]	63[b]	17[b]	36[b]	54[b]	26[b]	29[b]
Daly et al. (12)	2002-2009	30	53 (3y)	11 (37%)	8.1 (2.4-31.9)	45[c]	60[c]	10[c]	35[c]	50[c]	NR	60[c]
Sher et al. (13)	2004-2009	30	91 (2y)	2 (7%)	NR	26[c]	54[c]	0[c]	20[c]	43[c]	17[c]	77[c]
Chan et al. (17)	2005-2010	180	78 (2y)	38 (21%)	8 (2-39)	40[b]	68[b]	17[b]	34[b]	61[b]	5[b]	26[b]
Metcalfe et al. (20)	2007-2012	45	89/94[d] (2y)	2 (4%)	6 (2-34)	43[e]	73[e]	24[e]	38[e]	45[e]	38[e]	28[e]
Current report	2001-2012	289	79 (2y)	63 (22%)	4 (0-71)	44[c]	65[c]	7[c]	31[c]	41[c]	26[c]	41[c]

Abbreviations: PO-IMRT - post-operative intensity-modulated radiotherapy; OCC - oral cavity cancer; LRC - locoregional control; LRF - locoregional failure; RFS - recurrence free survival; ECE - extracapsular extension; PNI - perineural invasion; LVI - lymphovascular invasion; CRT - chemoradiation; NR - not reported
a - % of all patients in the study (n = 55, 49 with PO-IMRT to OCC)
b - % of all patients who had received PO-IMRT
c - % of only patients who had failed following PO-IMRT
d - local control was 89% and regional control 94% at 2 years
e - % of all patients in the study (n = 106, 45 with PO-IMRT to OCC)

Supplementary table 2: Published Reports Describing Patterns of Failure Following Post-Operative IMRT for Oral Cavity Cancer, Failure Characteristics

Author	LRF following initiation of PO-IMRT (n)	Spatial Classification Method	In Field Recurrences (n)	Marginal Recurrences (n)	Out Field Recurrences (n)
Yao et al. (9)	11	Binary volume overlap with CTV	10	0	1
Studer et al. (10)	NR	NR	NR	NR	NR
Gomez et al. (11)	6	Binary volume overlap with target volume	4[a]	0	0
Daly et al. (12)	11	Percentage volume overlap with 100% isodose CTV[b]	7	2	2
Sher et al. (13)	2	Percentage volume overlap with 95% isodose CTV[c]	2	0	0
Chan et al. (14)	38	Percentage volume overlap with 95% isodose CTV[d]	26	7	5
Metcalfe et al. (20)	2	Percentage volume overlap with 95% isodose CTV[d]	0	0	2
Current report	54	Spatial/dosimetric classification	30->A,10->C,2->G	2->B,1->D	9->E

Abbreviations: CTV – clinical tumor volume; NR – not reported
a - Study reported 4 local failures (all within treatment volume) and 2 regional failures (spatial classification not specified)
b - Per Chao et al. (20), >95% of recurrent tumor falling within CTV is infield, 20-95% is marginal, and <20% is outfield
c - Per Popovtzer et al. (21), >50% of recurrent disease present within 95% isodose line is infield and <50% is marginal
d - Per Dawson et al. (22) ≥95% of recurrence volume within 95% isodose of intended treatment dose is infield, 20 to <95% is marginal, and <20% is outfield
